# Comprehensive Characterization of Viral Diversity of Female Mosquitoes in Madagascar

**DOI:** 10.3390/v15091852

**Published:** 2023-08-31

**Authors:** Amal Bennouna, Michael Luciano Tantely, Vololoniaina Raharinosy, Soa Fy Andriamandimby, Thomas Bigot, Delphine Chrétien, Elise Jacquemet, Stevenn Volant, Sarah Temmam, Philippe Dussart, Vincent Lacoste, Romain Girod, Marc Eloit

**Affiliations:** 1 Pathogen Discovery Laboratory, Institut Pasteur, Université de Paris, 75015 Paris, France; amal.bennouna@pasteur.fr (A.B.); sarah.temmam@pasteur.fr (S.T.); 2 The WOAH (OIE) Collaborating Center for the Detection and Identification in Humans of Emerging Animal Pathogens, Institut Pasteur, Université Paris Cité, 75015 Paris, France; 3 Medical Entomology Unit, Institut Pasteur de Madagascar, Antananarivo 101 1274, Madagascar; lucinambi@pasteur.mg (M.L.T.);; 4 Virology Unit, Institut Pasteur de Madagascar, Antananarivo 101 1274, Madagascar; 5 Bioinformatics and Biostatistics Hub, Institut Pasteur, Université Paris Cité, 75015 Paris, France; 6 Ecole Nationale Vétérinaire d’Alfort, University of Paris-Est, 94700 Maisons-Alfort, France

**Keywords:** mosquitoes, *Culex*, *Aedes*, *Anopheles*, virome, metatranscriptomics, Madagascar

## Abstract

The diversity and circulation of arboviruses are not much studied in Madagascar. The fact is that arboviral emergences are rarely detected. The existing surveillance system primarily relies on serological detection and records only a few human infections annually. The city of Mahajanga, however, experienced a confirmed dengue fever epidemic in 2020 and 2021. This study aimed to characterize and analyze the virome of mosquitoes collected in Mahajanga, near patients with dengue-like syndromes to detect known and unknown viruses as well as investigate the factors contributing to the relative low circulation of arboviruses in the area. A total of 4280 mosquitoes representing at least 12 species from the *Aedes*, *Anopheles*, and *Culex* genera were collected during the dry and the rainy seasons from three sites, following an urbanization gradient. The virome analysis of 2192 female mosquitoes identified a diverse range of viral families and genera and revealed different patterns that are signatures of the influence of the mosquito genus or the season of collection on the composition and abundance of the virome. Despite the absence of known human or veterinary arboviruses, the identification and characterization of viral families, genera, and species in the mosquito virome contribute to our understanding of viral ecology and diversity within mosquito populations in Madagascar. This study serves as a foundation for ongoing surveillance efforts and provides a basis for the development of preventive strategies against various mosquito-borne viral diseases, including known arboviruses.

## 1. Introduction

Mosquitoes are hematophagous insects that are widely distributed, except in Antarctica. They inhabit distinct types of environments and are the main vectors of arboviruses in almost every part of the world. They can transmit pathogenic viruses, such as dengue virus (DENV), West Nile virus (WNV), Chikungunya virus (CHIKV), Zika virus (ZIKV), or Rift Valley fever virus (RVFV), and therefore pose a major public health concern worldwide. They are particularly present in biotopes with high temperatures and humidity such as tropical areas, where they encounter a wide diversity of vertebrates and plants and, consequently, of viruses [1]. Changes in their ecosystem, due to land use anthropization and global warming, contribute to the extension of their geographical distribution, leading to the colonization of novel areas. Hence, mosquito-borne diseases nowadays represent a global challenge for controlling their spread, not only in tropical regions but also in new and previously naïve areas [2].

Recently, metagenomics has proved to be a reliable approach for profiling vector-borne viruses. The development of next-generation sequencing (NGS) and new bioinformatics capacities have revealed a vast diversity of viruses in mosquitoes [3]. Metagenomic analysis of mosquitoes allow identifying all viruses present including arboviruses and viruses specific to insects (ISV), as well as viruses associated with their diet. Moreover, the recent characterization of mosquitoes’ viral communities from various locations around the world such as in Europe [4], the Caribbean [5], the USA [6] and China [7] have concluded that their virome was largely shaped by the mosquito species or genus, but the impact of mosquito species barrier on the virome composition is still largely unknown.

Over the past two decades, arboviruses such as DENV, CHIKV, and ZIKV have emerged as major zoonotic pathogens. Prior to their international emergence, these viruses were not recognized as major public health concerns or as causing significant diseases in Africa [8]. Furthermore, despite the extensive effort devoted to the study of viruses associated with human and animal diseases [9,10], little information is available to understand the diversity and transmission dynamics of arboviruses according to the ARBOCAT platform.

Madagascar is an island with an area of 587,041 square kilometers located in the southwest Indian Ocean, approximately 250 miles off the southeast coast of Africa. It is made up of five bioclimatic geographical regions. The east and northwest areas are characterized by a tropical climate facilitating efficient reproduction of mosquitoes. Moreover, Madagascar is home to numerous endemic plants and animals and is one of the richest countries in terms of vertebrate diversity [11,12]. It also exhibits a great diversity of mosquitoes with more than 235 referenced species, and more than 60 species known to be involved in pathogen, including arboviruses, transmission [13].

Although arboviral circulation was detected in Madagascar in the 1970s and 1980s [14], the island seems to have been relatively spared from arboviral human outbreaks, until 2006–2010, when dengue-like febrile syndromes were detected, with some of them positive for DENV, CHIKV, and RVFV [15,16,17,18,19]; however, all the criteria for an active arbovirus circulation were met.

Little information is available to understand the cause of this apparent low level of arboviral circulation. The national surveillance system, based on molecular and serological detection, reports only a few cases of human infections a year [20]. For example, in the western part of Madagascar, among 233 patients presenting with dengue-like illness, 203 were dengue-negative samples [20]. Additionally, the known serological cross-reactions among viruses belonging to the same viral family, for example, *Flaviviridae* [15], has raised questions about the potential presence of other arboviruses in Madagascar that may not have been detected or documented. Such arboviruses could silently circulate within the human population. Furthermore, Madagascar showcases various environments [20], which can be favorable for the acquisition of a vectorial competence by local mosquito species [21]. The epidemic risk seems, therefore, to be present. To obtain enough information on arbovirus diversity, exploring the virome existing in wild mosquitoes is necessary. The aim of this study was, therefore, to characterize and analyze viral diversity among mosquitoes collected in Madagascar, and to investigate the factors that shape virome composition. We performed a metatranscriptomics analysis to characterize the virome associated with different mosquito species collected in the city of Mahajanga along an urbanization gradient and during two seasons, and then compared the diversity and abundance of all viruses detected.

## 2. Materials and Methods

### 2.1. Study Sites

The seaport city of Mahajanga located in the northwest region of Madagascar was chosen as the study site based upon the high prevalence of dengue-like patients who tested negative for DENV and because of the presence of a well-defined gradient of urbanization and anthropization.

**Figure 1:**
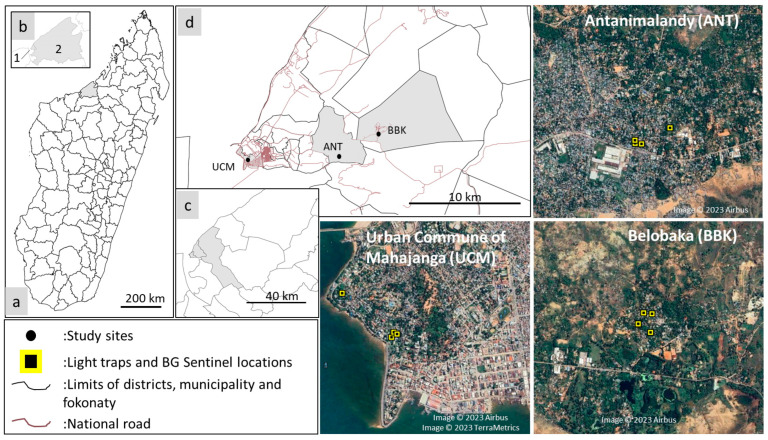
Location of the sampling districts (**a**,**b**), the three sampling municipalities (**c**,**d**), the sampling sites, and the traps used (satellite image). 1: Mahajanga I, 2: Mahajanga II, UCM: Urban Commune of Mahajanga, ANT: Antanimalandy, BBK: Belobaka.

Mosquito collections were conducted in three localities of the city of Mahajanga: (i) Urban Commune of Mahajanga (UCM), which constitutes the urban site, (ii) Antanimalandy (ANT), the peri-urban site, and (iii) Belobaka (BBK), the rural site (Figure 1, Table 1). Mosquito collections were performed at the end of the dry season (November 2021) and at the start of the rainy season (January 2022) to ensure an acceptable representativeness of the different mosquito species.

### 2.2. Mosquito Sampling, Identification, and Storage

Mosquitoes were sampled using CDC-light traps (LTs) (CDC miniature light trap, BioQuip Products, Inc., Rancho Dominguez, USA), with eight LTs per site in total, to assess the diversity and abundance of dusk, night, and dawn mosquitoes. Traps were positioned from 04:00 p.m. to 08:00 a.m. Human-odor-baited BG sentinel traps (BGs) (BioQuip Products, Rancho Dominguez, CA, USA), with eight BGs per site, were also used to determine the diversity and abundance of diurnal mosquitoes. They were positioned from 07:00 a.m. to 06:00 p.m. Three days and three nights of catches per site were performed. Outdoor resting mosquitoes were collected immediately after sunrise using a mouth aspirator from eight Muirhead–Thomson pit traps (MTPT), dug close to the houses where LTs and BGs were positioned. These three types of mosquito traps were placed near the houses of human cases presenting with dengue-like symptoms. A check of traps every four hours enabled the collection of live mosquitoes, and then we preserved viral RNA.

Mosquitoes were immediately morphologically identified in the field using a microscope positioned on a chilled table, and then counted, pooled according to the species, sex, and female status (fed or unfed), and stored in liquid nitrogen. Once in the laboratory, they were stored at −80 °C before RNA extraction.

### 2.3. RNA Extraction, NGS Libraries Construction, and Sequencing

Total RNAs were extracted from minipools comprising one to ten mosquitoes according to species, sex, and female engorgement status. Briefly, mosquitoes (comprising one to ten individuals) were crushed in 500 µL of PBS and centrifuged at 12,000× *g* at 4 °C for two minutes. A total of 100 µL of supernatant were mixed with 350 µL of RLT lysis buffer (Qiagen, Valencia, CA, USA) and 450 µL of absolute ethanol. Total RNA extraction was then conducted using the RNeasy mini kit (Qiagen, Valencia, CA, USA), according to the manufacturer’s instructions. RNA was eluted in 40 µL of RNase-free water.

Large pools containing up to 10 minipools (extracted RNA from mosquitoes) were constituted for a total of 51 pools containing a maximum of 100 mosquitoes each. Minipools were grouped by season, location, sex, species, and feeding status. RNA quality of the large pools was evaluated using the RNA pico chip (Agilent, Waldbronn, Germany) and an Agilent 2100 Bioanalyzer. NGS libraries were constructed using the SMARTer Stranded Total RNA-seq kit v3-Pico input mammalian kit (Clontech, Takara Bio, San Jose, CA, USA) according to the manufacturer’s instructions. NGS library profiles were validated using the dsDNA pico chip (Agilent, Waldbronn, Germany) and the Agilent Bioanalyzer and quantified with the dsDNA high-sensitivity kit (Invitrogen, Waltham, MA, USA) onto a Qubit 2.0 Fluotometer (Invitrogen, Waltham, MA, USA). Sequencing was carried out on an Illumina NextSeq 2000 sequencer in a paired-end 2 × 100 bp format to achieve approximately 50 million paired reads per library.

### 2.4. Virus Taxonomic Assignation

Raw sequencing reads were processed with Microseek, an in-house bioinformatics pipeline [21] that includes quality check and trimming, read normalization, de novo assembly, open reading frames (ORFs) prediction, and taxonomic assignation of contigs and singletons using (i) an exhaustive and curated viral sequence database RVDB-prot [22], itself derived from the nucleic Reference Virus DataBase (RVDB) [23], and (ii) generalist (NCBI/nr/nt) databases.

The pipeline is also based on the lowest common ancestor (LCA) of all the matches sharing the best score instead of randomly assigning to one of the best matches, which supports a more accurate overview of a sample content. For each taxonomic association, abundance is estimated by the number of corresponding nucleotides of the matching sequences; in other words, the sum of the lengths of the reads, or when it comes to contigs, the sum of the contig length multiplied by an estimate of its coverage, as provided by the assembler.

### 2.5. Statistical Analysis

To determine if we sequenced the whole diversity of the viral communities present per site, per mosquito genera, and species, rarefaction curves were constructed using the “rarecurve” function from the Vegan package [24]. The “Vegdist” function was employed to compute Jaccard similarity (based on binary indices) and Bray–Curtis dissimilarity (based on abundance-based species). These methods assess the level of community similarity and dissimilarity by considering the presence/absence (Jaccard) and abundance (Bray–Curtis) of shared species among sites, and species unique to each site as well site [25]. 

Differential abundance analysis was performed with SHAMAN [26] with default parameters. The count data matrix was normalized using the normalization method provided in the DESeq2 R package, and a variance stabilizing transformation (VST) was applied for visualization. To identify differentially abundant families among mosquito genera, a generalized linear model (GLM) was used. The GLM included season, site, and mosquito genera as main effects, as well as interactions between season and site. Resulting *p*-values were adjusted using the Benjamini and Hochberg procedure [27] to account for multiple comparisons. 

All tables describing viral families, genera, and species relative abundances were used for statistical analyses (principal coordinates analyses, differential analysis) in R software (v4.2.1) using ade4 [24] and Vegan [28] packages, and plots were generated with the ggplot2 package.

### 2.6. Phylogenetic Analysis

Phylogenetic reconstructions were conducted on the following genes: the conserved nonstructural RNA-dependent RNA polymerase (RdRP) for *Rhabdoviridae* and *Orthomyxoviridae* (PB1 only) and the complete polyprotein for the *Flaviviridae*. Naming of viral genomes was based on the geographic locations and host species from which genomes were generated. Other viral genomes used were obtained from NCBI GenBank database. 

An MAFFT (multiple alignment using fast Fourier transform) aligner was used to align the complete ORFs with other viral genus’ representative sequences under the auto-mode (version 7) [29]. Alignments were manually cured and the best amino acid substitution model that fitted the data were determined using the modified Akaike information criteria, which was LG + G for amino-acid *Rhabdoviridae*, *Flaviviridae*, and *Orthomyxoviridae* phylogenies. 

Based on the selected substitution model, phylogenetic trees were generated using the maximum likelihood (ML) method provided through the IQ-TREE program. The nodal support was assessed using the approximate Bayes parameter. Phylogenetic trees were visualized with the Interactive Tree Of Life tool (iTOL version 6) [30].

To generate identity matrices between *Guadeloupe Culex rhabdovirus*, complete genome nucleotides were aligned with MAFFT with the same parameters as those used for phylogenetic reconstructions, and matrices were generated using CLC Main Workbench v.21.0.4.

All virus genome sequences generated in this analysis have been deposited in GenBank under the Bioproject number PRJNA1004570.

## 3. Results

### 3.1. Mosquito Diversity across Sites and Seasons

A total of 4280 mosquitoes distributed among at least 12 species and belonging to the *Aedes*, *Anopheles*, and *Culex* genera were collected and identified from the three sites during the dry and rainy seasons (Figure 1, Table S1). *Culex quinquefsciatus* were the most abundant (n = 4003), followed by *Culex antennatus* (n = 126), *Culex tritaeniorhynchus* (n = 57), *Culex albopictus* (n = 50), and *Aedes aegypti* (n = 25), and the remaining species did not exceed 4 individuals (Table S1). More mosquitoes were sampled at the most urbanized UCM site (48.2%), followed by the peri-urban ANT site (34.9%) and the rural BBK site (16.9%). *Culex quinquefasciatus* was the predominant species, representing 93.5% (n = 4003) of the mosquitoes caught. The remaining *Culex* species (*Cx. antennatus*, *Cx. tritaeniorhynchus*, *Cx. univittatus*, and one unidentified *Culex*) represented only 1.5% (n = 63) of catches. Mosquitoes belonging to the *Aedes* genus (comprising five species and one unidentified *Aedes*) accounted for 85 and *Anopheles* (two species) for three females.

**Figure 2:**
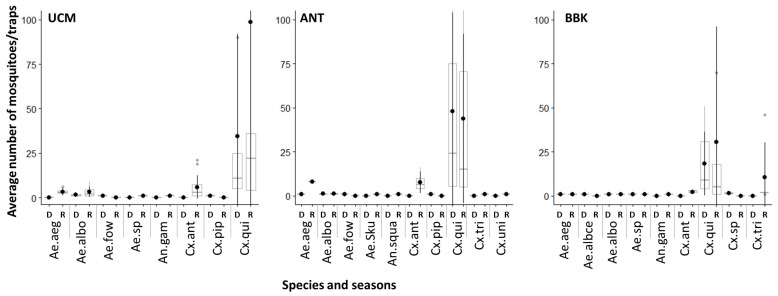
Variation of the number of mosquito collected per light trap during the dry (D) and rainy (R) seasons in the three sites of the city of Mahajanga. Ae.albo: *Aedes albopictus*, Ae.aeg: *Ae. aegypti*, Ae. fow: *Aedes fowleri*, Ae. sku: *Aedes* (*Skusea*), An. gam: *An. gambiae s.l.*; An. squa: *An. squamosus*, Cx. ant: *Cx. antennatus*, Cx. qui: *Cx. quinquefasciatus*, Cx. tri: *Cx. tritaeniorhynchus*, Cx. univ: *Cx. univittatus*. Circles refer to mean value.

No significant difference in the average number of mosquitoes was observed between LTs and BGs traps in general (Wilcoxon, *p* > 0.05) and between the different sites (Wilcoxon, *p* > 0.05). However, a higher density of mosquitoes was observed in LTs than in BGs during the rainy season (Wilcoxon, *p* < 0.05) (Table S1). During the dry and rainy seasons, *Cx. quinquefasciatus* was the most abundant species (Kruskal–Wallis, *p* < 0.05), except in BBK, where no significant difference was observed in the abundance of each species during rainy season (Kruskal–Wallis, *p* = 1) (Figure 2).

**Figure 3:**
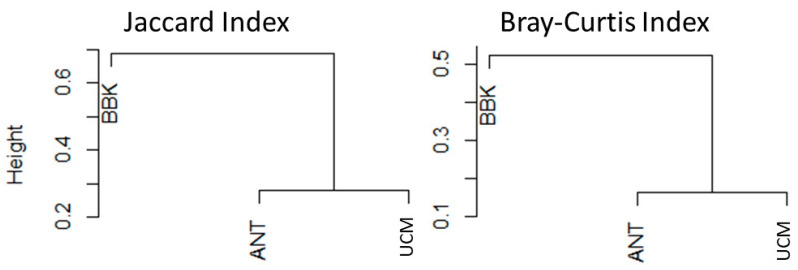
Hierarchical clustering illustrating species abundance similarity among female mosquitoes captured at the three sites of the city of Mahajanga (UCM = 8 species, ANT = 10 species, BBK = 10 species) (Jaccard index = presence/absence; Bray–Curtis index = abundance).

The hierarchical clustering analysis based on Jaccard and Bray–Curtis indices revealed distinct patterns among the study sites. On the one hand, the diversity of mosquito species of the urban site UCM and the peri-urban site ANT formed a closely related cluster (Figure 3), indicating a high similarity between these sites. On the other hand, the rural site BBK displayed a different clustering pattern, suggesting a greater dissimilarity between sites presenting different levels or urbanization (and subsequent anthropization). In fact, among the mosquito species encountered, *Aedes aegypti*, *Ae. albopictus*, *Cx. antennatus*, and *Cx. quinquefasciatus* were present at all sites, testifying to their wide distribution. *Aedes fowleri* and *Cx. pipiens* were common species observed in both UCM and ANT, while *Aedes* (*Skusea*) sp. and *Cx. tritateniorhynchus* were present in ANT and BBK. Finally, *An. gambiae* was found in both UCM and BBK, while *An. squamosus*/*cydippis* and *Cx. univittatus* were exclusively present in ANT, and *Ae. albocephalus* in BBK.

### 3.2. Viral Families Diversity by Site, Mosquitoes Genus, and Species

It is important to note that our initial sequencing batch involved testing male mosquitoes, but no discernible distinctions were observed between male and female mosquito viromes. Consequently, we decided to concentrate on female mosquitoes due to their exclusive role as vectors of pathogens through hematophagous behavior.

Of the 4280 mosquitoes collected, 2192 female mosquitoes (51.2% of the total caught) were combined in 51 pools and used for metatranscriptomics analysis (Table S2). The remaining 48.8% of total caught mosquitoes were not tested because they all belonged to the *Culex quinquefasciatus* species. These 51 pools represented three mosquito genera and nine species over two seasons and three sites. Most of the reads were classified as eukaryotic (mosquito) and bacterial genomes, while the abundance of viral genomes ranged from 0.003% to 0.090%.

**Figure 4:**
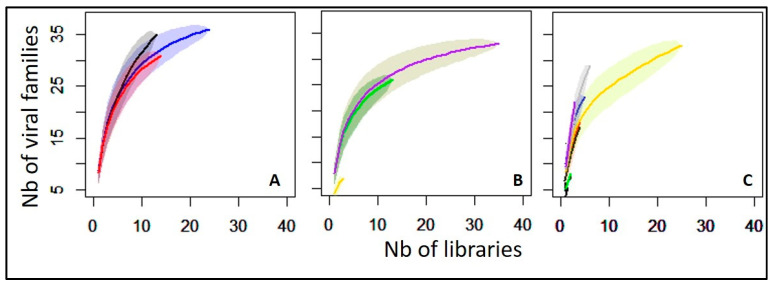
Rarefaction curve for (**A**) viral families as function of sites (blue = UCM, red = ANT, black = BBK), for (**B**) viral families as function of mosquito genera (yellow = *Anopheles*, green = *Aedes*, purple = *Culex*), and for (**C**) viral families as function of mosquito species (red = *Ae. aegypti*, black = *Ae. albopictus*, blue = *Aedes* sp., green = *An. gambiae* s.l, gold = *Cx. quinquefasciatus*, purple = *Cx. tritaeniorhynchus*, gray = *Cx. antennatus*). *Anopheles squamosus*/*cydippis* and *Cx. pipiens* were not analyzed due to the single library used in this study.

The viral hits obtained from the analysis were distributed into 33 viral families and nine unclassified taxa. Rarefaction analysis revealed to what extent the whole variability of viral taxons was captured regarding the sites, mosquito genus, and species (Figure 4). At the virus family level, the curve reached a plateau for the UCM site, showing a relatively comprehensive sampling of viral diversity in this location, compared to the peri-urban ANT or the rural BBK sites. Of note, the BBK site demonstrated a higher richness than both UCM and ANT while the curve continued to rise, implying that additional sampling and sequencing efforts are necessary to capture the full extent of viral diversity at this site (Figure 4A). When looking at the mosquito genus level (Figure 4B), the curve related to the *Culex* genus reached the plateau, meaning that a relatively comprehensive identification of viral diversity within this genus was achieved. Conversely, curves for *Aedes* and *Anopheles* genera continued to increase, suggesting that additional efforts are required to fully uncover the viral diversity associated with these two mosquito genera. This is in line with the high number of *Culex* mosquitoes collected on the three sites, conversely to the *Aedes* and *Anopheles* genera, which was confirmed by the analysis performed depicting viral richness at the species level (Figure 4C). The characterization of the global virus diversity was not achieved for *Ae. aegypti*, *Ae. albopictus*, *Ae. sp* (mixture of *Ae. fowleri*, *Ae* (Skusea) sp. and other unidentified *Aedes* species), *An. squamosus*, *Cx. antennatus*, and *Cx. tritaeniorhynchus*. These species seem to harbor a greater diversity of viral families that are yet to be fully characterized.

### 3.3. Overview of Virus Diversity Carried by Mosquitoes

**Figure 5:**
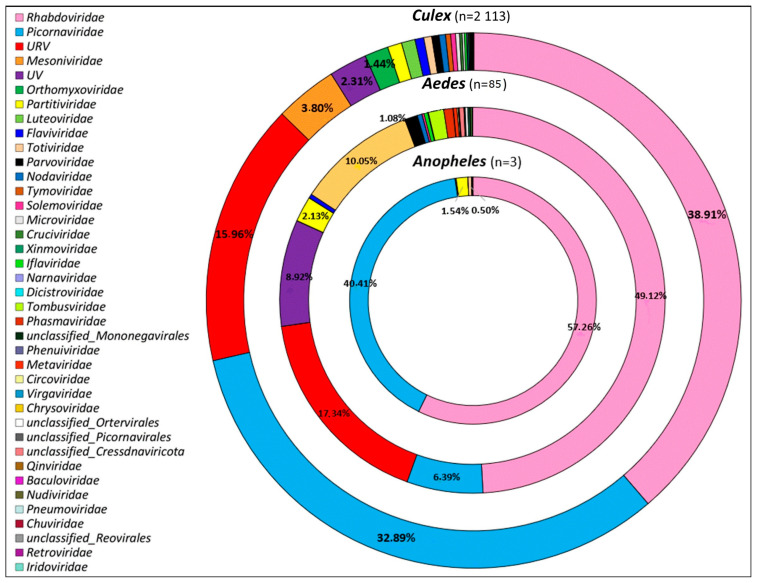
Overview of viral families identified in the three mosquito genera (*Culex*, *Aedes*, and *Anopheles*) collected in the city of Mahajanga, Madagascar. URV: unclassified RNA viruses, UV: unclassified viruses.

Among *Culex* mosquitoes (n = 2113), which constituted the most abundant genus, a substantial proportion of viral sequences belonged to several prominent viral families, including *Rhabdoviridae*, followed by *Picornaviridae*, and “unclassified RNA viruses” (Figure 5). Other viral families present, albeit in smaller proportions, included *Mesoniviridae*, “unclassified viruses”, Orthomyxoviridae, *Partitiviridae*, and *Luteoviridae*. Similarly, for *Aedes* mosquitoes (n = 85), *Rhabdoviridae* was the most abundant viral family, followed by *Picornaviridae* and “unclassified RNA viruses”. Other viral families present included “unclassified Viruses” and *Partitiviridae*. The remaining viral families were of minimal proportions. Finally, for *Anopheles* mosquitoes (n = 3), the majority of viral sequences belonged to the *Rhabdoviridae*, *Picornaviridae*, and *Partitiviridae* families (Figure 5). Interestingly, these findings indicated that *Rhabdoviridae* and *Picornaviridae* were present and common to all three genera, albeit in different proportions, suggesting that the virome composition may be influenced by the mosquito genus. Conversely, each mosquito genus was also infected by unique viral families, with *Culex* having the most diverse range of viral families compared to *Aedes* and *Anopheles*.

**Figure 6:**
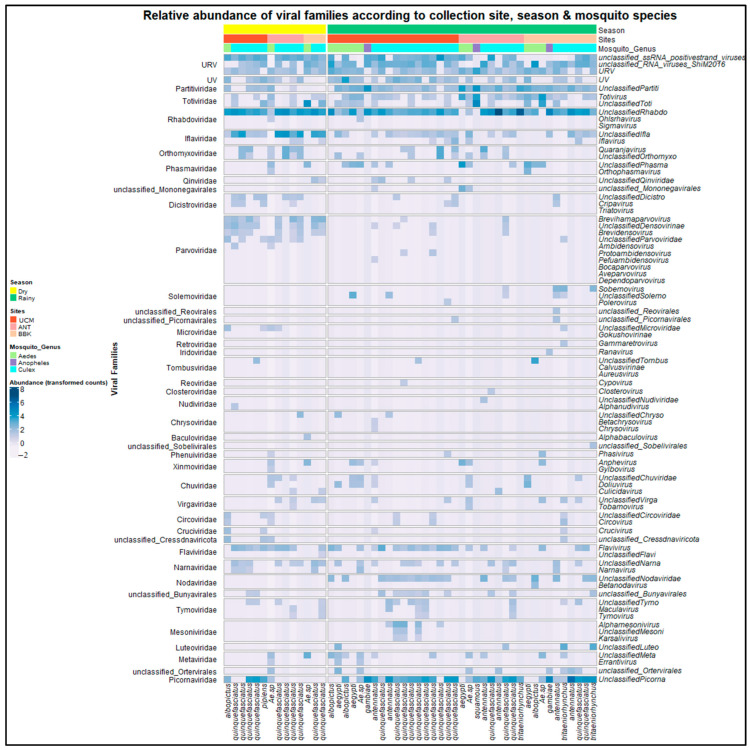
Relative abundance of viral genera according to the season, sites, and genus of collected mosquitoes. The names of 33 viral families are listed in the left-hand column and the names of 44 viral genera are in the right-hand column of the heatmap. Mosquito species are shown at the bottom. Seasons, sites, and mosquito genera are shown at the top. The colors represent the transformed counts according to sequence abundance, from light blue (low abundance) to dark blue (high abundance). URV: unclassified RNA viruses, UV: unclassified viruses.

At the viral genus level, at least 44 viral taxons were identified in addition to unclassified viruses (Figure 6). The heatmap revealed the relative abundances of viral genera according to the mosquito species, seasons, and collection sites, providing a comprehensive overview of the virome composition. While only a few viruses were classified into recognized genera (*Iflaviviridae*/*Iflavirus*, *Orthomyxoviridae*/*Quaranjavirus* and *Mesoniviridae*/*Alphamesonivirus*), the majority belonged to unclassified genera in their respective families: unclassified *Rhabdoviridae*, unclassified *Picornaviridae*, unclassified *Iflaviviridae*, unclassified *Totiviridae*, unclassified *Partitiviridae*, unclassified *Luteoviridae*, etc. (Figure 6).

When looking at the species level, our investigation yielded a comprehensive collection of 281 distinct viruses associated with the viral families and genera described above (Table S3). However, to ensure a high degree of confidence in our taxonomic assignations, we applied a stringent criterion, retaining only those viral sequences that exhibited a minimum of 90% amino acid identity to the last common ancestor. Following this threshold, we narrowed down the list to 62 viral species (Figure S1), with distinct pattern of abundance and diversity through seasons, sites, and mosquito species. *Guadeloupe Culex rhabdovirus* and *Grenada mosquito rhabdovirus 1* (*Rhabdoviridae*), were the most abundant viral entities in our samples. Following closely in terms of abundance were *Xiang Yun picorna-like virus 4* (*Picornaviridae*), *Culex pipiens-associated Tunisia virus* (URV), and *Culex flavivirus* (*Flaviviridae*) (Figure S1).

**Figure 7:**
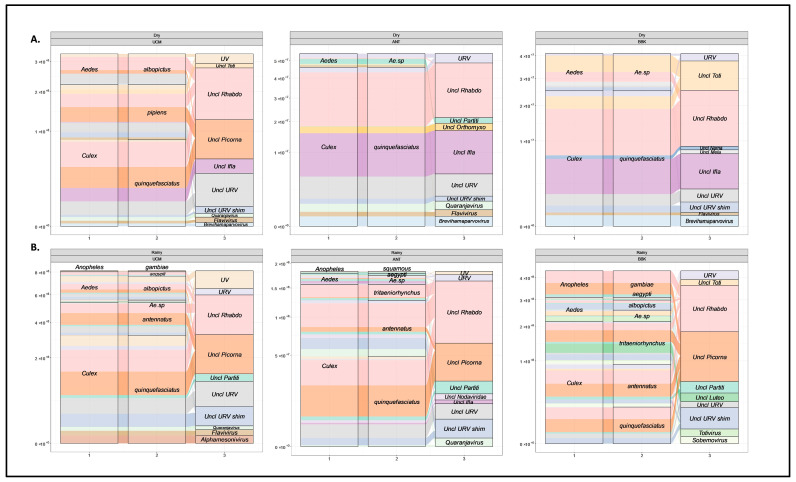
Alluvial plot showing the ten most abundant viral genera for all collection sites (UCM, ANT, and BBK) and the two seasons (**A**) dry and (**B**) rainy. They represent the total sum of normalized counts found for each viral genus (right nodes + color by viral family name) and their repartition by mosquito species (middle nodes) and genus (left nodes).

To further investigate the specific viral genera contributing to the differences observed between the viromes of mosquito species and genera, we employed an alluvial plot visualization approach focusing on the ten most abundant viral genera (Figure 7) and the virus species having more than 90% of amino acid identity (Figure S2). This analysis provided a clear representation of the major contributors to the virome composition, and highlighted the prominent viral groups shaping the virome profiles of mosquito populations at each site and season. This approach effectively captured and visualized the main viral associations within the mosquito virome, providing valuable information on the dominant viral components. By examining the alluvial plots (Figure 7) and (Figure S2), we observed distinct assortments of viral genera and species depending on various factors (season, collection site, and mosquito species/genus). On the one hand, certain viral genera and species were consistently present in specific mosquito species at different sites and seasons (unclassified *Rhabdoviridae*|*Guadeloupe Culex rhabdovirus*, *Grenada mosquito rhabdovirus 1*), indicating their potential association with certain mosquito species. On the other hand, variations in the distribution of viral genera and species between sites and seasons were also observed, revealing dynamic changes in the virome composition likely impacted by ecological and seasonal factors or by the level of urbanization (unclassified *Orthomyxoviridae*|*Culex pipiens orthomyxo-like virus*) (Figure 7 and Figure S2).

### 3.4. Phylogenetic Analysis of Selected Viral Species

We strategically chose to characterize three viruses that not only belong to families known for their arboviral potential (*Rhabdoviridae*, *Flaviviridae*, and *Orthomyxoviridae*), but are also among the most dominant within our sampled mosquito populations.

**Figure 8:**
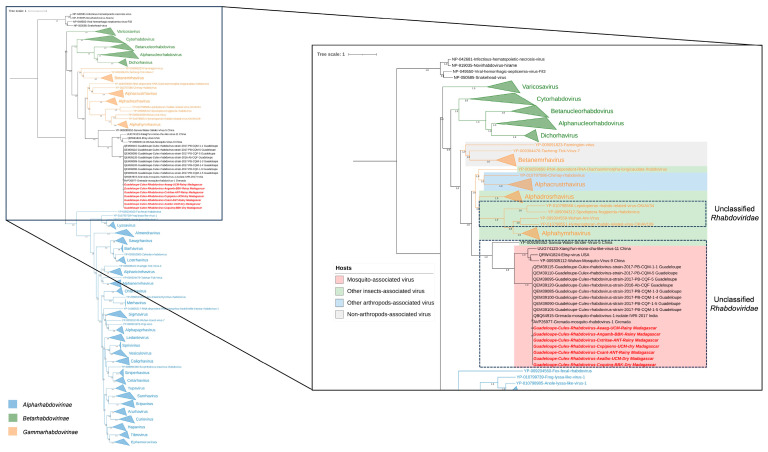
Phylogenetic relationship of the RNA-dependent RNA polymerase domain of *Guadeloupe Culex rhabdovirus* identified in Madagascar (in red) with other mosquito-associated viruses (red), other insect-associated viruses (green), other arthropod-associated viruses (blue), and non-arthropod-associated viruses (gray).

The majority of reads belonging to the *Rhabdoviridae* family were assigned to *Guadeloupe Culex rhabdovirus* (GCRV) and *Grenada mosquito rhabdovirus 1* (GMRV1) in all mosquito species, depending on the gene explored. Complete genomes were obtained from our sequencing data, and presented amino-acid identities over 99.8% to each other and over 99% to GCRV and GMRV1, suggesting that the sequences from Madagascar, Guadeloupe, India, and Grenada are strains of the same viral species. In fact, phylogenetic analyses performed on the RNA-dependent RNA polymerase placed the malagacy GCRV in the same clade, including GCRV from Guadeloupe as well as GMRV1 from India and Grenada (Figure 8). These viruses are clustering with unclassified *Rhabdoviridae* and are closely related to other mosquito-related viruses such as *Wuhan mosquito virus 9* and *Elisy virus.* Interestingly, our analysis showed that the same virus with over 99% nucleotide and amino-acid identity was found across all sampled conditions, regardless of season, site of collection, or mosquito-host-related variation (Figure S3). Prevalence data further support the significance of malagacy GCRV within this context. Among the 51 samples tested, the virus was consistently detected in a substantial proportion. Specifically, it was found in 41 out of the 51 samples, indicating a prevalence rate of approximately 80.39%. This high prevalence underscores the ubiquity of malagacy GCRV and its persistent presence across various conditions.

**Figure 9:**
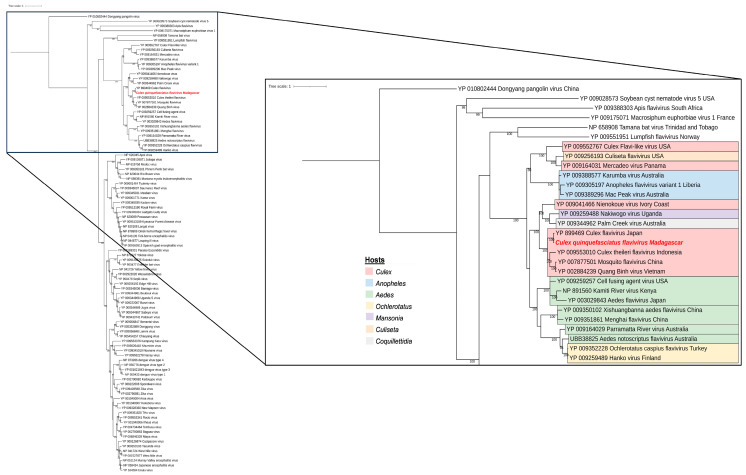
Phylogenetic relationship of the polyprotein genome of *Culex flavivirus* identified in Madagascar (in red) with insect-specific viruses.

A large proportion of reads belonging to the *Flaviviridae* family were assigned to *Culex flavivirus* (CxFV). This virus was found only in *Culex* samples and the complete genome was obtained from sequencing contigs and reads, presenting amino-acid identities ranging from 98% to 100% when compared to the reference sequence, depending on the protein considered. Phylogenetic analyses completed on the full polyprotein placed the malagacy CxFV in the same clade including other Culex-specific flaviviruses (Figure 9), which were closely related to other mosquito-species-specific flaviviruses. Moreover, our sequence seems to be the same as the strain isolated from *Culex pipiens* in Japan in 2003 [31]. It is important to highlight that the malagacy CxFV displayed a notable prevalence within *Culex* samples. Among the 35 samples of *Culex* mosquitoes that were tested, the virus was detected in 25 of these samples. This indicates a prevalence rate of approximately 71%, which underscores the significant presence of CxFV within *Culex* mosquitos in Madagascar.

**Figure 10:**
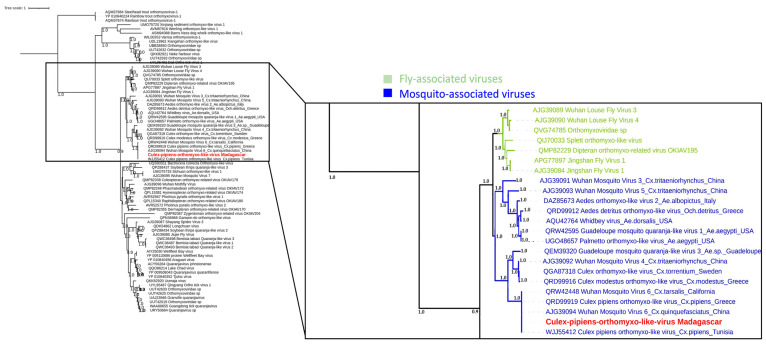
Phylogenetic relationship of the PB1 protein (segment 1) of *Culex pipiens* orthomyxo-like virus identified in Madagascar (in red) with other mosquito and fly associated viruses.

Another virus linked to the *Orthomyxoviridae* family was *Culex pipiens* orthomyxo-like virus. The malagacy strain was identified in *Culex quinquefasciatus* mosquitoes with a prevalence rate of approximately 9.8% (5 samples among 51 tested). Sequencing reads and contigs presented an amino-acid identity ranging from 98% to 100% when compared to the reference sequence, depending on the segment considered (PB1, PB2, and NP). Phylogenetic analyses performed on the segment 2 (PB1) placed our virus in the same clade as other mosquito-associated viruses, which is also closely related to other fly-associated viruses (Figure 10). Also, our sequence is the exact same as *Culex pipiens orthomyxo-like virus* isolated from *Culex pipiens* in Tunisia and Greece, as shown in Figure 10. 

### 3.5. Factors That Shape Mosquito Viral Communities in Mahajanga

**Figure 11:**
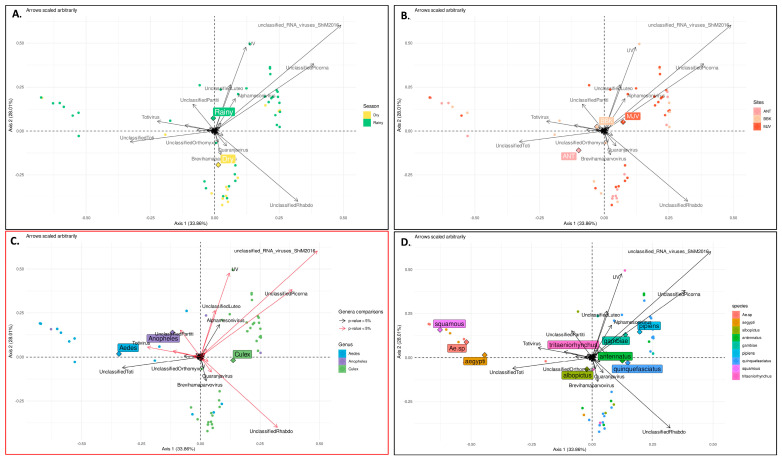
Principal coordinates analysis (PCoA) conducted on normalized counts to visualize differences between season (**A**), sites (**B**), mosquito genus (**C**), and species (**D**). The first two axes capturing 61% of variability within our data are represented. For genus comparison, viral genera that were found significant by differential analysis are shown in red. Circles represent projected values for each samples; for each group (detailed in legend) the mean point is represented as a square; arrows represent the direction of gradient of abundance for each taxa.

To further characterize the factors shaping the composition of the mosquito viromes, principal coordinate analysis (PCoA) was performed (Figure 11). The results confirmed that mosquito genus significantly influenced virome composition, since a clear clustering was observed between *Aedes* sp., *Culex* sp., and *Anopheles* sp. viral communities (Figure 11C). However, PCoA revealed no significant effect of other factors such as the season (Figure 11A), collection sites (from urban to rural) (Figure 11B), the feeding status, or the mosquito species (Figure 11D), even though subtle variations were observed, such as, for example, for the *Parvoviridae* family, which exhibited higher abundance during the dry season (Figure 6). Nevertheless, these variations were not statistically significant.

Differential analysis, used to specifically determine which viral genera presented significant differences in abundance between mosquito genera, showed which specific viral taxa contributed to the observed variations in virome composition (Figure S4). For example, the Flavivirus genus was significantly more represented in *Anopheles* sp. mosquitoes than in *Aedes* sp. or *Culex* sp. mosquitoes, while the genus *Quaranjavirus* was significantly more represented in *Aedes* compared to *Culex* mosquitoes. Overall, the main viral taxons that contributed to the specificity of *Aedes*, *Culex*, and *Anopheles* sp. viromes were unclassified *Phasmaviridae*, unclassified *Metaviridae*, unclassified viruses, unclassified *Iflaviridae*, unclassified *Rhabdoviridae*, and unclassified *Narnaviridae* (Figure S4).

**Figure 12:**
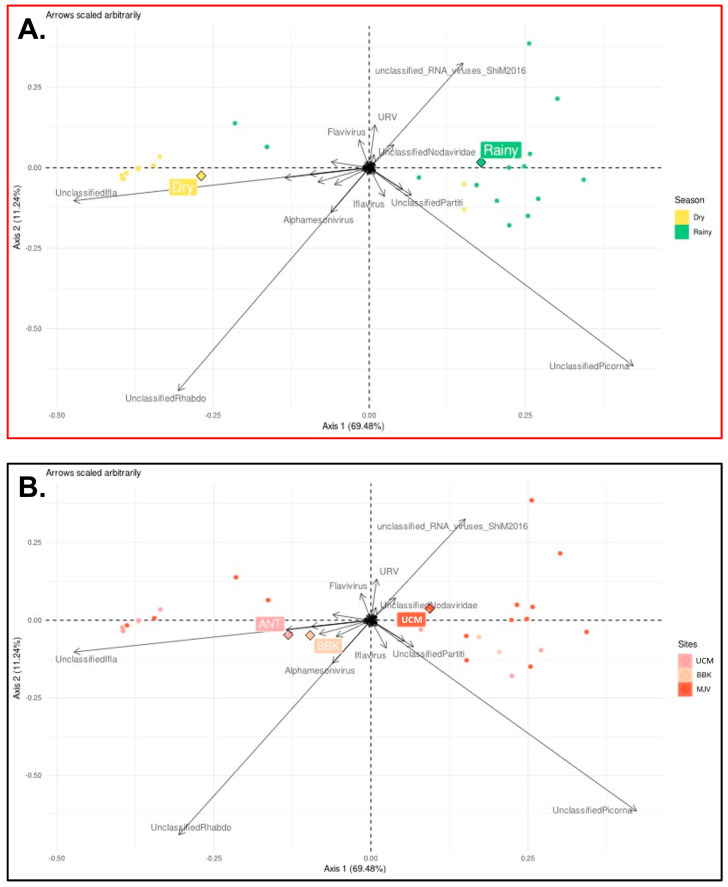
Principal coordinates analysis (PCoA) conducted on normalized counts of viral genera identified in *Culex quinquefasciatus* mosquitoes to visualize differences between seasons (**A**) and sites (**B**). The first two axes capturing 81% of variability within our data are represented. Circles represent projected values for each samples; for each group (detailed in legend) the mean point is represented as a square; arrows represent the direction of gradient of abundance for each.

To investigate the interaction between seasons and collection sites, a sufficient number of samples is required. In this study, only the *Culex quinquefasciatus* sample size was sufficient to conduct such an analysis. We therefore carried out a specific PCoA and conducted additional differential analyses to study the potential impact of these factors on the virome composition of *Cx. quinquefasciatus*. The GLM model included season and site as main effects, as well as interactions between season and site. The results obtained from this analysis revealed a clear effect of season on the virome composition of *Cx. quinquefasciatus* mosquitoes (Figure 12A), while the impact of collection sites appeared to be comparatively less pronounced (Figure 12B). Viruses belonging to unclassified Densovirinae, unclassified *Iflaviridae* and “unclassified ssRNA positive strand viruses” taxa were more abundant during the dry season, whereas those belonging to unclassified Luteoviridae, unclassified *Nodaviridae*, unclassified *Picornaviridae*, unclassified *Phasmaviridae*, unclassified *Metaviridae*, unclassified *Partitiviridae*, and “unclassified viruses” taxa were significantly more present during the rainy season (Figure S5).

## 4. Discussion

Lessons learned from the COVID-19 pandemic have heightened the importance of exploring known and unknown viruses in wildlife to prevent potential outbreaks. This includes the development of reliable detection tools for viruses and the identification of potential vectors. Mosquito-borne diseases represent a significant global public health concern and pose substantial health risks. Understanding the composition, abundance, and viral diversity of mosquito populations is essential for assessing disease transmission dynamics and developing effective control strategies. To our knowledge, this study represents the first systematic and extensive inventory of viruses carried by mosquitoes in Madagascar. This pioneering research provides a solid foundation for a more accurate assessment of mosquito management, vector competence, and the associated risk of mosquito-borne diseases. By thoroughly examining the viral composition, abundance, and diversity within mosquito populations, we can gain valuable insights into disease transmission dynamics and facilitate the development of targeted control measures.

In the framework of this study, the sampling effort yielded a diverse range of mosquito species belonging to three genera: *Culex*, *Aedes*, and *Anopheles*, with *Culex* being the most abundant genus and *Culex quinquefasciatus* the predominant species identified. *Culex quinquefasciatus* is the main vector of West Nile virus in Madagascar and is widely distributed across the country, showing a preference for urban environments and various biotopes (sewage, tires, plastic containers, metal drums, etc.) [13]. The higher abundance of *Culex* genus compared to other genera, observed here, is consistent with previous reports [29]. One study highlighted the abundance of *Culex* genus and the differences in mosquito abundance between urban, peri-urban, and rural areas [13]. In contrast, *Aedes* sp. mosquitoes that are considered the main vectors of several endemic viruses in the area, such as DENV, were less frequent in our sampling. Additionally, *Cx. quinquefasciatus*, *Ae. Aegypti*, and *Ae. albopictus* are known as bioindicators of urbanization [13,15], and the decrease in the abundance of (at least) *Cx. quinquefasciatus* from the urban (UCM) to the rural site (BBK) confirmed the presence of a gradient of urbanization between these study sites. Also, the presence of *An. squamosus/cydippis* in the peri-urban site (ANT) was expected, due to its occurrence in the peripheral areas of urban regions [13]. The remaining species are typically associated with rural areas [13] and were, indeed, found in the BBK rural site.

With regard to the seasonal dynamics of mosquito species, greater abundance of mosquito species was observed during the rainy season in all urban, peri-urban, and rural sites, as previously reported in Madagascar [13]. However, this marked trend was mainly observed for *Cx. quinquefasciatus*, which was the most abundant species. It should be noted that the presence of artificial and polluted breeding sites, often found in urban and peri-urban areas, provides favorable conditions for the development of this species [13], not to mention that temporary rain pools and floodplains represent suitable breeding sites as well. 

Overall, the impact of urbanization on mosquito diversity and abundance can be discussed in the context of contrasting patterns observed among the study sites. The highest abundance of *Cx. quinquefasciatus* in urban areas, the decrease in abundance from urban to rural sites, and the differences in species composition suggest a potential influence of the urbanization level on mosquito populations. Our findings align with previous studies bringing out the association between urban environments, human activities, and mosquito abundances [31]. It is important to consider that the observed patterns could also be influenced by other factors such as sampling methods, collection efforts, and potential biases in mosquito trapping. However, the consistency of the results with the known ecological characteristics of the study sites supports the validity of our findings. They are further supported by the rarefaction analysis, which provided a perspective on viral diversity and supports the influence of urbanization, geography, and mosquito species composition. 

Further research and investigations are needed to explore these hypotheses in more detail, considering additional environmental factors such as breeding site characteristics and human–mosquito interactions. Understanding the underlying mechanisms shaping the composition of mosquito populations across different biotopes will contribute to our knowledge of mosquito ecology and to the development of effective vector control strategies and arboviral surveillance programs.

Our entomological investigation was performed in areas where high prevalence of dengue-like patients not confirmed as DENV-positive cases assumes the circulation of other arboviruses and the presence of associated mosquito vectors [13]. The twelve species collected during this study are each already reported to be involved in transmission of at least five arboviruses in the world [14]. Analysis of the viral communities infecting mosquito species provides valuable insight into the virome and vector interactions that can influence mosquito vectorial competence. We present here the first comprehensive characterization of the viral communities infecting mosquitoes in Madagascar. The predominant viral families identified were *Rhabdoviridae* and *Picornaviridae*, which is consistent with previous studies [32,33]. Other families were present in smaller proportions, such as *Mesoniviridae*, *Orthomyxoviridae*, *Partitiviridae*, and *Luteoviridae*. These findings support the notion that mosquitoes can harbor a rich and varied virome [34,35,36].

Importantly, the virome composition exhibited significant variations between mosquito genera. The PCoA demonstrated distinct clustering of viral communities among *Aedes* sp., *Culex* sp., and *Anopheles* sp., indicating that different mosquito genera harbor unique viral communities in terms of diversity and abundance (this was confirmed by differential analysis). However, several factors such as season, collection site, and mosquito species had a relatively minor role in shaping the virome composition, as their effects were not statistically significant. Although subtle variations in virome composition associated with season, site, or mosquito species were observed for some viral families, further exploration is needed to determine their significance. These results suggest that the mosquito genus plays a predominant role in determining the composition of the virome, as reported elsewhere [37], highlighting the importance of considering host-specific factors in virome studies. This observation emphasizes that different mosquito genera may have distinct viral associations and potential interactions (as shown by alluvial plots, for instance), which can influence the transmission dynamics of viruses.

Of particular interest is the occurrence of specific viral taxa, particularly in *Culex quinquefasciatus*, across seasons and sites, implying their potential importance within the mosquito virome. PCoA analysis, combined with an additional differential analysis, demonstrated a clear significant effect of the season on the presence and abundance of specific viruses. In other words, these viruses are continuously present among mosquito populations across the two seasons, but their abundance vary. The origin of this increase of abundance, linked to the rainy season, can be related simply to a higher abundance of mosquito hosts, or can be hypothesized by at least two explanations. If we suppose that these viruses are part of the core virome, their continuous detection means that they persist throughout the year. Therefore, their increasing abundance observed during the rainy season may either be linked to an external (i.e., environmental) supply of viruses occurring during the development stage of the mosquito, or may be due to an increase of replication of these viruses within the mosquito host. Conversely, the impact of collection sites (and the subsequent impact of urbanization and anthropization) on virome diversity and composition appeared to be less pronounced than other factors. This may be attributed to factors such as the homogeneity in mosquito populations across the studied sites or other site-specific variables that were not adequately captured in our analyses.

It is important to elaborate on the concept of the core virome, which refers to a set of viruses consistently associated with a specific host group (such as *Cx. quinquefasciatus*). These core viromes, along with other components of the microbiome, can have a significant impact on biological processes of the host [38,39]. The core virome differs from transient viruses, such as arboviruses, which are acquired through meals and may have a more dynamic presence. It should be noted that even though these “nontransmitted” viruses may not directly cause diseases in humans and animals, they can still impact vector competence of arboviruses. Characterizing the diversity and composition of the mosquito virome, including the core virome, is therefore crucial to understanding the broader viral ecology and dynamics within mosquito populations from Madagascar.

In conclusion, the absence of detected known human or veterinary arboviruses does not necessarily indicate the absence of arboviral activity in the studied area. Several factors could contribute to the absence of arbovirus detection, such as the low prevalence of arboviruses within mosquito populations in the region, the timing of sample collection, and even some limitations related to our study and the sensitivity of the detection methods employed (metatransciptomic approach can vary regarding the efficiency of RNA extraction, library preparation, sequencing, and bioinformatic analyses). This underscores the need for continued sampling efforts, particularly targeting *Aedes* and *Anopheles* mosquitoes, to actively monitor viral activity within mosquito populations. In addition to monitoring the circulation of known human or veterinary arboviruses, a comprehensive characterization of the core virome and other viral agents is crucial. Exploring their specific roles and potential implications for vector competence and disease transmission requires in-depth investigations, including viral genomic characterization, host–virus interaction studies, and detailed environmental monitoring. Furthermore, employing agnostic approaches to analyze samples collected from patients exhibiting arbovirus-like symptoms with unknown etiology would serve as a complementary strategy. Continuous and comprehensive surveillance, along with extensive research endeavors, will enhance our understanding of viral dynamics and facilitate the development of effective strategies for the control and prevention of mosquito-borne diseases.

## 5. Conclusions

Even in the absence of known human or veterinary arboviruses, characterization of the mosquito virome and identification of viral families and genera contribute to our awareness of viral ecology and diversity within mosquito populations in Madagascar. This study provides a foundation for ongoing surveillance efforts and for the development of preventive strategies against a range of mosquito-borne viral disease, including known arboviruses.

## Figures and Tables

**Table 1:** Description of the three sites where mosquito collections were performed.

Districts	Villages Names	Description of Landscape
Mahajanga I	Urban Commune of Mahajanga 15′43′10″80″ S 46′18′20″25″ E	35 m above sea level (asl); the historical neighborhood of the city, with large modern cement houses/building made of cement occupying almost 100% of the area.
Mahajanga I	Antanimalandy 15′42′53″22″ S 46′21′32″97″ E	17 m asl; made up of cement houses, wooden houses, and sheet metal houses, which occupy almost 50% of the area.
Mahajanga II	Belobaka 15′42′12″21″ S 46′23′34″24″ E	15 m asl; the majority of habitations are made of sheet metal house and occupy 30% of the area. A lot of mango trees, swamp, and rice fields are observed.

## Data Availability

Raw data associated with characterized viruses were deposited into the NCBI/SRA database and are available under the Bioproject number PRJNA1004570. The genome sequences of characterized viruses were deposited under accession numbers SAMN36946652, SAMN36946653, SAMN36946654, SAMN36946655, SAMN36946656, SAMN36946657, SAMN36946658, SAMN36946659, SAMN36946660, and SAMN36946661.

## Supplementary Materials

The following supporting information can be downloaded at: https://www.mdpi.com/article/10.3390/v15091852/s1. Table S1: Mosquito collection number per site, season and trap; Table S2: Mosquito sequencing data generations; Table S3: List of viruses detected in mosquito collected in Madagascar; Figure S1: Relative abundance of viral species according to the season, sites, and genus of collected mosquitoes; Figure S2: Alluvial plot showing the 62 viral species with >90% amino-acid identity to their known reference sequences, for all collection sites (UCM, ANT, and BBK) and the two seasons (A) dry and (B) rainy; Figure S3: Pairwise nucleotide identity percentage across *Guadeloupe Culex rhabdovirus* from different seasons, sites, and mosquito host species of Madagascar; Figure S4: Log2 fold change for the comparisons of *Culex*, *Aedes*, and *Anopheles* in the differential analysis conducted. The color corresponds to significant viral genera; Figure S5: Log2 fold change for the comparisons of dry/rainy seasons in the differential analysis conducted on *Culex quinquefasciatus* mosquitoes. Each panel represents a different site studied. The color corresponds to significant viral genera.
