# Supplementary material for: Comprehensive Characterization of Viral Diversity of Female Mosquitoes in Madagascar
Source: Viruses. 2023 Aug 31;15(9):1852. doi: 10.3390/v15091852 (PMC10537517; doi:10.3390/v15091852)
Supplement: Supplementary file 1 [file viruses-15-01852-s001.zip › viruses-2520628-supplementary-figures.pdf]

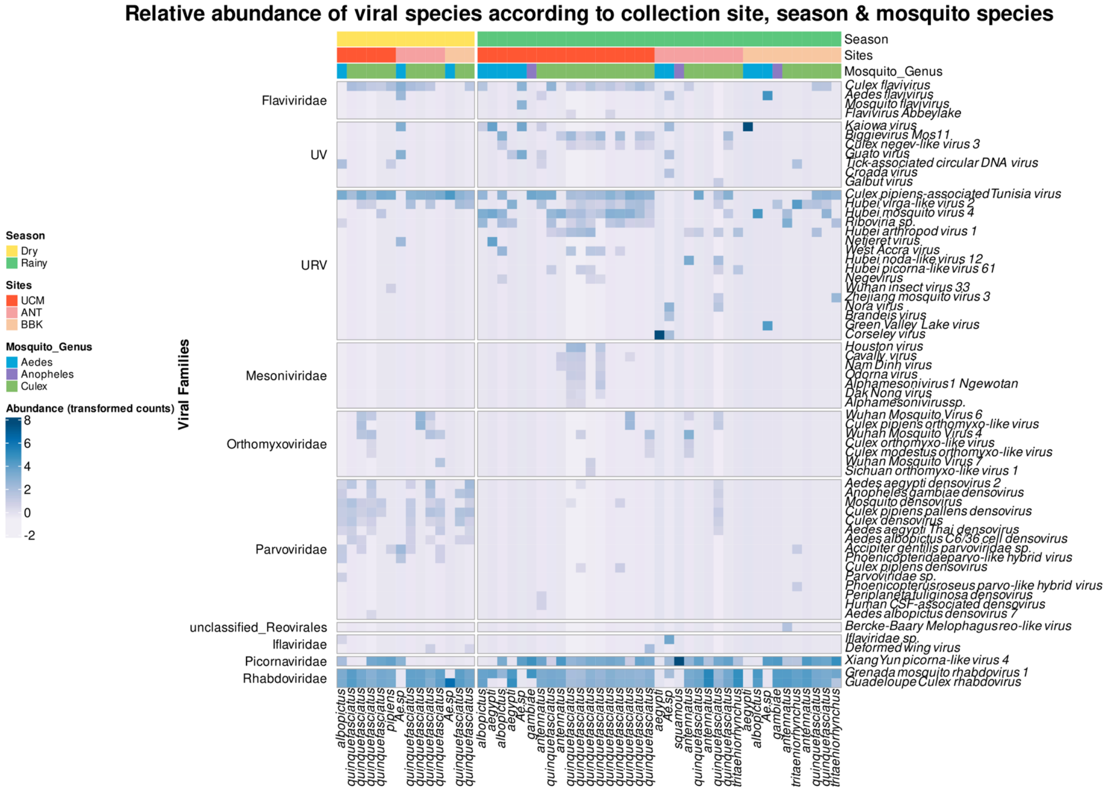

Figure S1: Relative abundance of viral species according to the season, sites, and genus of collected mosquitoes.

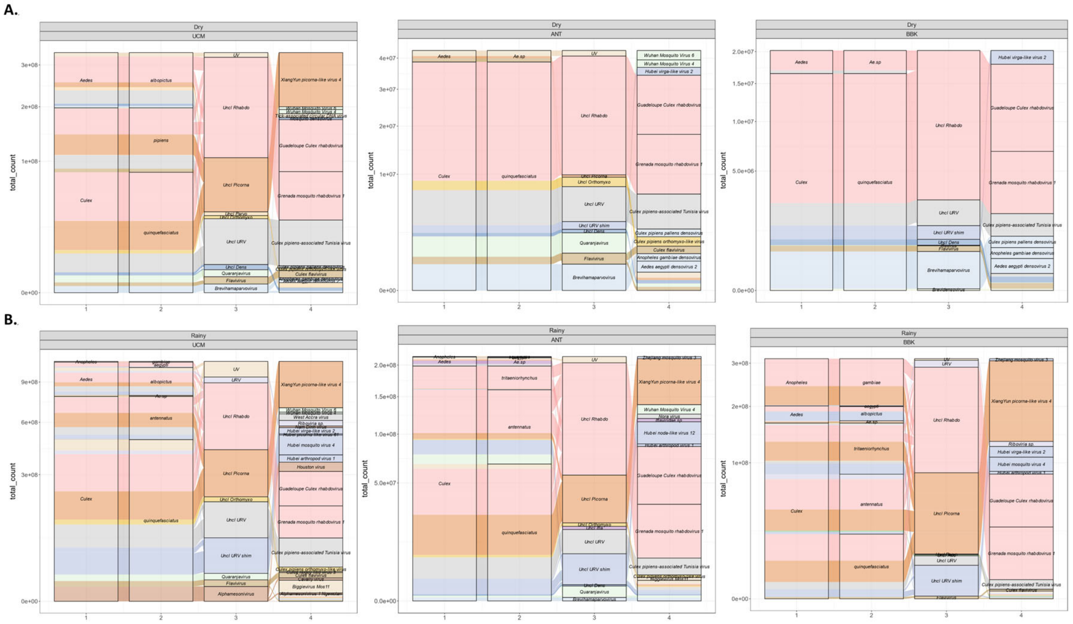

Figure S2: Alluvial plot showing the 62 viral species with >90% amino-acid identity to their known reference sequences, for all collection sites (UCM, ANT, and BBK) and the two seasons (A) dry and (B) rainy.

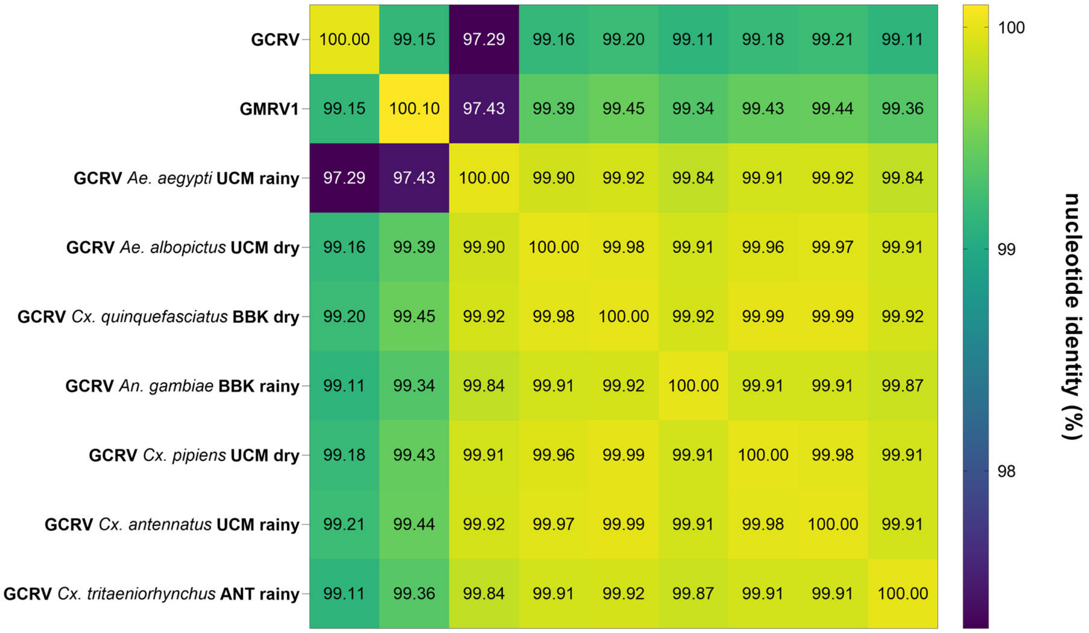

Figure S3: Pairwise nucleotide identity percentage across *Guadeloupe Culex rhabdovirus* from different seasons, sites, and mosquito host species of Madagascar.

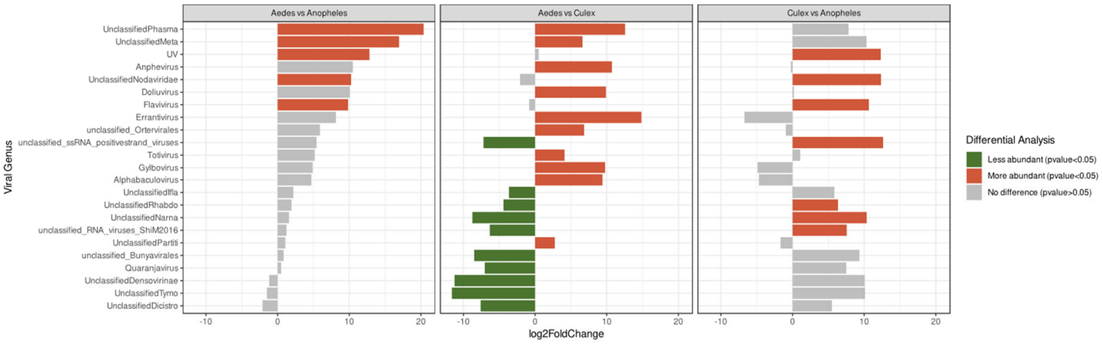

Figure S4: Log2 fold change for the comparisons of *Culex*, *Aedes*, and *Anopheles* in the differential analysis conducted. The color corresponds to significant viral genera.

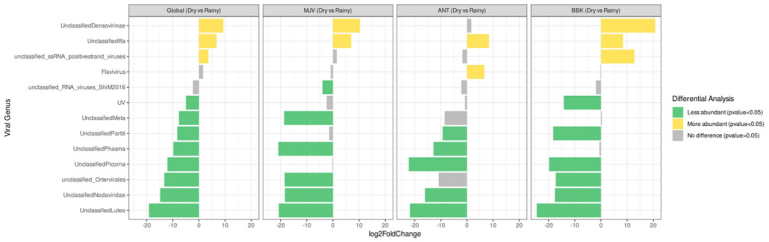

Figure S5: Log2 fold change for the comparisons of dry/rainy seasons in the differential analysis conducted on *Culex quinquefasciatus* mosquitoes. Each panel represents a different site studied. The color corresponds to significant viral genera.
